# The Expansion of the Homœopathic Mind

**Published:** 1881-09

**Authors:** 


The Expansion of the Homœopathic Mind is well illustrated by the course of our esteemed contemporary, the *New York Medical* (*née Homœopathic*) *Times.* Since it dropped the title "homœopathic" it has been studying to exclude that objectionable word altogether from its columns. And it has even been referring to the American Institute of Homœopathy as the "Institute," adding, of course, the annual announcement that it is the "oldest national medical organization in the country." The *Times* still thinks us trammelled by the absurd traditions of the past, and we think the *Times* and its contributors ought to learn pathology. But progress is affecting everything. It has affected our esteemed contemporary, and we will yet see it print reports of our Pathological Society. The editors announce that they will practise hereafter as regular physicians. The rule (not law) of similars will be used in selecting remedies only when such rule seems advisable. The homœopathic materia medica, we are told, is largely an imaginative work ; it contains a frightful bulk of non-essentials and unproved statements. They believe in no medical dogmas, and assert that medical knowledge and skill are the only tests of the physician. Homœopathy, as a distinct school of medicine, has ceased to exist among educated men. This is what the *Times* says, or means, and we can say no more.

